# Sweetmeats

**Published:** 1874-04

**Authors:** 


### SWEETMEATS.

IT ought to be known that pies, pastries, puddings, and sweet cakes of every description, if well made, and sugar candies, if pure, are not only not injurious to the health, but promote digestion, and thus give increased nutriment to the system.

These statements were made many years ago, and have been repeated from time to time in the pages of this journal. Every child ever born luxuriates in sweet things. Perhaps no reader can point out a person who does not delight in sweets, unless there is some disease in the system. Let us reason about it.

Medical men know that if babies were prevented from having sweets in their food they would die in a very short time. It is the sweet of their food that keeps them warm. The belief is almost universal that sugar and sweetmeats injure the teeth. If you put a spoonful of sugar in a cup of tea it disappears—it is entirely dissolved ; if it is eaten it is dissolved with the saliva, and is passed into the stomach in a minute or two ; nothing whatever is left in the mouth, or about the teeth, or between the teeth. There is not even the taste of sweetness on the tongue or teeth five minutes after being taken into the mouth ; it is all in a dissolved state in the stomach, a foot or two away.

There is no vegetable or fruit which does not contain sugar. A loaf of wheat bread is healthful if sugar is added to it ; it is then much more nutritious, and if the Creator has combined the element of sweetness in all that grows out of the earth suitable for human food, it must be because the element of sweetness is necessary to the wants of the system.

Adams and Liebig, the most able analyzers of human food, have investigated the subject closely and faithfully in its chemical relations, and have arrived at the fact that whatever of sweetness there is in our food makes it the more nutritious, and that food somewhat difficult of digestion is made more digestible by sweetness; and no doubt it was this observed fact, without knowing the reason of it, which has led to the almost universal practice in civilized life of having something sweet after the principal meal of the day, in the form of desserts, all of which are sweetened. It has also been found that all nuts used at the table have an oil, which also promotes digestion. The only way in which sweets in desserts can injure is in connection with their not being properly prepared or being used too freely. We should look at things in the light of reason, of facts, and a sound judgment. A short examination of the subject, chemically, will assist in forming a truthful opinion. It was found, by Dr. Bridges Adams, that when persons feed on beef killed when poor and tired on a march, very little nourishment and strength were imparted ; but when fat cattle were killed when not fatigued, there was an amazing difference in vigor of body and mental elasticity. But when fat cattle could not be had, and it was lean or none, by adding sugar in some form hunger was appeased. Infants take nourishment very often. It is because the sweets in it cause a rapid assimilation of the food. No wonder they have an inappeasable instinct for sweets; it would be a cruelty, a murder, to deprive them of what is necessary to life itself. The gastric or stomach juice contains hydrochloric acid. It is this which dissolves the food and prepares it for yielding its nourishment to the system. There is another acid which has a similar effect, and its elements are found in various kinds of food. This is lactic acid, which is formed when sugar comes in contact with the saliva in the mouth. So far from sugar injuring the teeth it hardens them, makes them more solid, and lasting, and beautiful, and gives strength and durability to the bones, because they are formed mainly of the phosphate of lime, which is found largely in all the grains, such as oats, wheat and corn ; and, wherever lactic acid finds the phosphate of lime, it dissolves it more perfectly, hence provides a larger supply of this indispensable material to the human body than if sugar were not taken. From these statements it is clear that in whatever vehicle sugar is carried into the system it does good, is healthful, is nutritious, is necessary, whether it be in pies, pastries, puddings or sugar candy. If these things are used in excess they will cause mischief, and so will bread and butter and beefsteak. If bread is badly baked, if roast beef is burned to a crisp, they become injurious, and so will a sodden cake, a doughy plum pudding or a sour mince pie. There is no more beautiful, attractive and healthful dessert than a stick of pure candy. Sweets, to be healthful, should not only be taken in moderation, but just before, during, or immediately after a regular meal, and at no other time, as a habit, for it is then they are needed to promote the more complete and perfect digestion of the food. It is taking sweetmeats between meals, at short intervals, in excessive quantities, which does injury, as would any form of human nutriment, however healthful and beneficial, in rational, moderate quantities.

